# Modeling Photo-Bleaching Kinetics to Create High Resolution Maps of Rod Rhodopsin in the Human Retina

**DOI:** 10.1371/journal.pone.0131881

**Published:** 2015-07-21

**Authors:** Martin Ehler, Julia Dobrosotskaya, Denise Cunningham, Wai T. Wong, Emily Y. Chew, Wojtek Czaja, Robert F. Bonner

**Affiliations:** 1 Faculty of Mathematics, University of Vienna, Vienna, Austria; 2 Department of Mathematics, Applied Mathematics, and Statistics, Case Western Reserve University, Cleveland, OH, United States of America; 3 Office of the Clinical Director, National Eye Institute, National Institutes of Health, Bethesda, MD, United States of America; 4 Unit on Neuron-Glia Interactions, National Eye Institute, National Institutes of Health, Bethesda, MD, United States of America; 5 Division of Epidemiology and Clinical Applications, National Eye Institute, National Institutes of Health, Bethesda, MD, United States of America; 6 Department of Mathematics, University of Maryland, College Park, MD, United States of America; 7 Section on Medical Biophysics, National Institute of Child Health and Human Development, National Institutes of Health, Bethesda, MD, United States of America; Medical University Graz, AUSTRIA

## Abstract

We introduce and describe a novel non-invasive in-vivo method for mapping local rod rhodopsin distribution in the human retina over a 30-degree field. Our approach is based on analyzing the brightening of detected lipofuscin autofluorescence within small pixel clusters in registered imaging sequences taken with a commercial 488*nm* confocal scanning laser ophthalmoscope (cSLO) over a 1 minute period. We modeled the kinetics of rhodopsin bleaching by applying variational optimization techniques from applied mathematics. The physical model and the numerical analysis with its implementation are outlined in detail. This new technique enables the creation of spatial maps of the retinal rhodopsin and retinal pigment epithelium (RPE) bisretinoid distribution with an ≈ 50*μm* resolution.

## 1 Introduction

Currently, many retinal diseases are clinically evaluated by subjective examination of retinal images [1] and lower resolution visual field testing [2]. Improved quantitative analysis in clinics should increase understanding of mechanisms of early retinal disease progression by mapping local changes in bleachable rhodopsin within the rod outer segments and bisretinoid levels within the underlying RPE. Localized reduction in bleachable rhodopsin may be an early indicator of local visual cycle defects and future rod loss, whereas local increases in bisretinoid fluorescence within the RPE has been associated with RPE stress and early sign of loss of function [3–6]. Thus, reliable clinical noninvasive maps of bleachable rhodopsin distribution in conjunction with RPE autofluorescence may provide a means to assess early intervention and disease prevention strategies. In the present work we develop the methodology to quantify rod rhodopsin using standard clinical cSLO autofluorescence imaging and analysis enabled by state-of-the-art mathematical techniques. The paper is addressed to both, applied mathematicians and vision scientists. The latter can familiarize themselves with computational details that can significantly improve quantitative results. Applied mathematicians may better comprehend the biomedical aspects enabling future design of customized mathematical tools beyond rhodopsin measurements. Our efforts aim at improving the understanding between both communities to enable further synergies. Acronyms that may not be well-known to one or the other community are explained in Table 1. Readers who prefer to avoid the mathematical details can skip Sections 3 and 4.

**Table 1:** Biomedical and mathematical abbreviations and terms.

*AF*	autofluorescence
apical	an anatomical term of location
bisretinoid	fluorescent reaction products created in the photoreceptors
carotenoids	organic pigments, intake through the diet
chromophores	molecules absorbing light in a wavelength depending fashion
cSLO	confocal scanning laser ophthalmoscope (clinical imaging device)
*D*	optical tissue density
*D*_*MP*_	optical macular pigment density
*I*	radiant power of the excitation light
*L*	bleaching constant, reciprocal of “photosensitivity”
lipofuscin	fluorophore in the RPE
lysomal	lysosomes are cellular organelles, cell’s waste disposal system
lutein, zeaxanthin	compounds of macular pigments
*K*	time constant of rhodopsin regeneration
*k*_*MP*_	relative extinction coefficient of macular pigment
*k*_*rh*_	relative extinction coefficient of rhodopsin
macula, fovea	center and center dip of the retina associated to central vision
macular pigment	pigment concentrated in the macula
maculopathy	pathological condition of the macula
melanin	pigment in the RPE
melanosome	organelle containing melanin
melanolipofuscin	granule exhibiting properties of both melanosomes and lipofuscin
nasal horizontal meridian	centered horizontal line from the fovea in the direction of the nose
*Ops*	unbound opsin
perifovea	peripheral region of the macula
*R*	fraction of rhodopsin
retinal, retinol	chemical forms of vitamin A
retinoids	class of compounds related to vitamin A, includes retinol and retinal
reticular druse	certain morphological features observed in retinal pathology
rod rhodopsin	chromophore located within rods in the retina
RPE	retinal pigment epithelium (single cell layer in the retina)
Stargardt’s macular dystrophy	certain form of juvenile macular degeneration
superior vertical meridian	centered vertical line above the fovea
vermillion (filter)	opaque red pigment, used as rod-protecting filters in sunglasses
11-cis, all-trans	conformational state of the bounds in a molecule
Λ, Λ_*j*_	excitation wavelengths
*λ*, *λ*_*j*_	emission wavelengths
Φ	fluorescence efficiency of lipofuscin
*ϱ*_*rh*_	rhodopsin density
Euler-Lagrange equations	solutions yield potential local minimizers of optimization problem
Ginzburg-Landau energy	functional used for image inpainting here
gradient descent	numerical optimization algorithm
Hessian	matrix of second derivatives
image inpainting	filling in missing parts of an image by using surrounding pixels
ODE	ordinary differential equation
semi-explicit finite differences	a class of methods for solving differential equations numerically
∂_*r*_	differential operator (“partial derivative along *r*”)
*τ*	artificial time variable used within the gradient descent method

The retina is a multi-layer neural tissue, uniquely suited for noninvasive optical imaging due to the evolutionary design of the eye and its ocular media. Located just above the choroid, the RPE is a single cell layer that nourishes overlying photoreceptors. Noninvasive autofluorescence imaging of bisretinoids in the RPE and of absorbing molecules in the overlying retina offers the possibility to sensitively monitor early changes in retinal function and early pathophysiology.

Localized rod photoreceptor losses have been observed in post mortem histology of age-related maculopathy, and the spatial rod distribution during normal aging has been characterized in humans [7–10]. Rod rhodopsin bleaching has been widely observed [5, 11–18], and degraded dark adaptation during aging and disease progression has been attributed to reduction in visual cycle regeneration of cis-retinal and rod rhodopsin. Thus, reduced bleachable rhodopsin and pigmentary changes may locally reflect early RPE dysfunction [19–25]. Although the biophysical model of dark adaptation and rod rhodopsin bleaching was introduced in [5, 11, 15, 18, 26], attempts of rod rhodopsin quantification in humans from retinal bleaching using standard clinical instruments has only recently been reported [27, 28] and not yet reduced to a routine clinical method. Bleaching kinetics are dependent on the actual retinal irradiance of the rhodopsin within the rod outer segments and therefore slowed by overlying retinal chromophores, such as macular pigments and hemoglobin, and by lens pigments. Lens absorption of blue laser light uniformly reduces retinal irradiance over the entire field of view in the cSLO. On the other hand, hemoglobin within retinal vessels effectively masks both the bleaching of underlying rhodopsin and RPE autofluorescence, which prevents analysis of pixels beneath visible retinal vessels. The macular pigments, lutein and the related carotenoid zeaxanthin, are concentrated within the photoreceptor nerve fibers of the fovea and reduce irradiance at 488*nm* at both the rod outer segment and RPE levels to an increasing degree as one approaches the center of the fovea [29–31]. Therefore, current bleaching and regeneration models must be extended to incorporate macular pigment contributions, and in Section 2 we shall combine the underlying biophysical models. Melanin within the RPE is more uniformly distributed [32] and is behind the photoreceptors, so that it reduces only the irradiance at the level of RPE lipofuscin and not the rod rhodopsin. Consequently, variations of melanin in the RPE and choroid within the central macula only reduce the autofluorescence amplitudes and not the rate of rhodopsin bleaching. To integrate measured autofluorescence images into the developed model, we shall use state-of-the-art variational analysis techniques from applied mathematics. The rhodopsin distribution maps are computed by optimization procedures outlined in Section 3. As a postprocessing step described in Section 4, we detect retinal vessels through image analysis and refine mathematical image inpainting methods to derive a spatial rod rhodopsin map of the human retina, in which retinal vessels are removed. Section 5 is a brief summary of our numerical approach studied in the Sections 3 and 4. We present examples and compare our rhodopsin maps with the typical distribution of rod photoreceptors in Section 6. Results are discussed in Section 7 and conclusion are given in Section 8.

## 2 Biophysical models

This section is dedicated to present a biophysical foundation of rod rhodopsin quantification from standard imaging devices. After highlighting the use of standard clinical instruments to measure retinal autofluorescence, we shall recall the rhodopsin bleaching and regeneration model in [5], refine the macular pigment model from [33], and combine both models. We shall also describe the underlying clinical protocol.

### 2.1 Retinal autofluorescence imaging

As light penetrates the retina, it is largely unscattered and only locally absorbed by retinal chromophores (first, hemoglobin within large retinal vessels, then macular pigments largely in the photoreceptor axons, then the unbleached opsins within the photoreceptor outer segments) before reaching the lipofuscin granules in the RPE. The emitted fluorescence from unoxidized lipofuscin bisretinoids can be excited by a range of visible wavelengths (blue to yellow) to yield long-wavelength emission in the red, which is detected noninvasively by the fluorescence imaging camera. Let Λ and *λ* be the excitation and emission wavelengths, respectively, and *AF*(Λ, *λ*) denotes the measured autofluorescence. The Beer-Lambert law for the double-path yields

$$\begin{array}{c} \text{AF}\left(\Lambda ,\lambda \right)=I\left(\Lambda \right)\Phi \left(\Lambda ,\lambda \right)e^{-(D(\Lambda )+D(\lambda ))}, \end{array}$$ (1)

where *D*(Λ) and *D*(*λ*) are the optical densities of the underlying tissue at wavelengths Λ and *λ*, respectively, Φ(Λ, *λ*) is the fluorescence efficiency of lipofuscin, and *I*(Λ) is the radiant power of the excitation light.

We use two different imaging techniques: First, to measure macular pigment, we developed autofluorescence imaging of the human retina at varying emission and excitation wavelengths by modifying standard fundus cameras [34]. Secondly, to subsequently quantify rod rhodopsin, we record images at a commercial cSLO camera (Heidelberg Retinal Angiograph 4.0) that delivers an average of ≈ 3*μW*/*mm* ^2^ at 488*nm* by rapidly scanning a small laser beam (10*μm*) over the retina. The intensity of excitation in the cSLO is > 100-fold less than in the fundus camera so that each cSLO image is acquired over a 100*ms* scan with an incremental rhodopsin bleaching (≈ 1%). After ≈ 40*s* of cSLO imaging, the rhodopsin is completely bleached (> 98%), and its initial attenuation of the excitation light at 488*nm* is removed. To map the rod rhodopsin from the magnitude of the brightening of the autofluorescence in registered movies, we shall study the quantitative models of bleaching and macular pigment absorption in more detail.

### 2.2 Rod rhodopsin models

#### 2.2.1 Bleaching model

In a dark-adapted retina the rod opsin is predominantly in its bound form with 11-cis retinal, i.e., rhodopsin. Rhodopsin strongly absorbs green-blue (≈ 500*nm*) light, which photo-isomerizes its bond cis-retinal to all-trans-retinal inducing protein conformational changes and subsequent photoreception signaling. These various downstream forms lose the strong green-blue absorbance, hence rhodopsin bleaching refers to its conversion by light to these non-absorbing forms [5, 15, 18].

If we start with a dark-adapted retina, then the intensity of the local autofluorescence, *AF*, within a small circle of pixels should increase as the rhodopsin bleaches (becomes transparent to the 488*nm* exciting laser light). The unbleached rhodopsin in the photoreceptor layer initially attenuates the lipofuscin excitation by ≈ 50% outside the central fovea, and the excitation light reaching the RPE then progressively increases as the overlying rhodopsin is locally bleached. Thus, we need to incorporate the time course in Eq (1), so that we have

$$\begin{array}{c} \text{AF}\left(\Lambda ,\lambda ,t\right)=I\left(\Lambda \right)\Phi \left(\Lambda ,\lambda \right)e^{-(D(\Lambda ,t)+D(\lambda ,t))}, \end{array}$$

where we assume a constant radiation power *I*(Λ). The optical density changes over time, and contributing chromophores are macular pigments in the fovea and rhodopsin in the perifoveal region. There is, however, a region within the fovea, where both are present at a significant density. The macular pigment contribution was not accounted for in [5, 15, 18]. Here, we explicitly model the signal attenuation caused by macular pigment absorption to obtain rod rhodopsin maps covering the entire macula. Therefore, we obtain

$$\begin{array}{c} D\left(\Lambda ,t\right)+D\left(\lambda ,t\right)=D_{MP}\left(\Lambda \right)+D_{MP}\left(\lambda \right)+D_{rh}\left(\Lambda ,t\right)+D_{rh}\left(\lambda ,t\right), \end{array}$$

where *D*_*rh*_ and *D*_*MP*_ denote the optical density of present rhodopsin and macular pigments, respectively. If *R*(*t*) denotes the fraction of rhodopsin remaining at time *t*, then we obtain

$$\begin{array}{c} D_{rh}\left(\Lambda ,t\right)+D_{rh}\left(\lambda ,t\right)=\left(D_{rh}\left(\Lambda ,0\right)+D_{rh}\left(\lambda ,0\right)\right)R\left(t\right), \end{array}$$

where *R*(0) = 1 corresponds to the dark adapted retina and *D*_*rh*_(Λ,0)+*D*_*rh*_(*λ*, 0) is the double path optical density of rhodopsin present at time *t* = 0. If *ϱ*_*rh*_(0) denotes the physical density of present rhodopsin at time *t* = 0 and its molar extinction coefficient is *k*_*rh*_, then we have

$$\begin{array}{c} D_{rh}\left(\Lambda ,0\right)+D_{rh}\left(\lambda ,0\right)=\varrho_{rh}\left(0\right)\left(k_{rh}\left(\Lambda \right)+k_{rh}\left(\lambda \right)\right), \end{array}$$

which yields

$$\begin{array}{c} \text{AF}\left(\Lambda ,\lambda ,t\right)=I\left(\Lambda \right)\Phi \left(\Lambda ,\lambda \right)e^{-(D_{MP}\left(\Lambda \right)+D_{MP}\left(\lambda \right)+\varrho_{rh}\left(0\right)\left(k_{rh}\left(\Lambda \right)+k_{rh}\left(\lambda \right)\right)R\left(t\right))}. \end{array}$$ (2)

In order to determine *ϱ*_*rh*_(0), we still need to specify *R*(*t*).

#### 2.2.2 Regeneration model

We use the model developed in [5, 15] to specify the fraction of rhodopsin remaining at time *t* due to the rates of rhodopsin photoactivation and of its regeneration by the visual cycle. The visual cycle is a complex process that transports the released all-trans-retinoid to the underlying retinal pigment epithelium, there converts it back to 11-cis-retinal and returns it to the rod opsin. The normal time constant for visual cycle regeneration of rhodopsin increases with age from ≈ 700*sec* to ≈ 1000*sec* and is much greater than the rate of rhodopsin bleaching in autofluorescence imaging with 488*nm* cSLOs.

To specify *R*(*t*), the fraction of rhodopsin remaining at time *t*, we need to model the regeneration process, for which we started with Michaelis-Menten kinetics of the production of cis-retinal from cis-retinol. The change in the fraction of unbound opsin *Ops*(*t*) = 1 − *R*(*t*) is proportional to the 11-cis retinal concentration that binds to opsin,

$$\begin{array}{c} S+E⇆[ES]⇆E+P, \end{array}$$

where *S* is 11-cis retinol, *E* is 11-cis RDH (RDH5), and *P* is 11-cis retinal. The fractional change of unbound opsin satisfies

$$\begin{array}{c} \frac{\partial }{\partial t}Ops\left(t\right)=-kP\left(t\right)Ops\left(t\right). \end{array}$$

Thus, when rhodopsin is bleached by a steady light of illuminance *I*, its regeneration resembles a first-order reaction $\frac{1-R(t)}{K},$ where *K* is the time constant. Therefore, the fractional change of the rhodopsin concentration satisfies

$$\begin{array}{c} \frac{d}{dt}R\left(t\right)=-\frac{IR(t)}{L}+\frac{1-R(t)}{K}, \end{array}$$ (3)

where *L* is a “bleaching constant” that corresponds to the reciprocal of “photosensitivity” and has been measured by retinal densitometry [35, 36]. Eq (3) has the analytical solution

$$\begin{array}{c} R\left(t\right)=\frac{\frac{L}{K}}{I+\frac{L}{K}}+\frac{I}{I+\frac{L}{K}}e^{-\left(1+\frac{KI}{L}\right)\frac{t}{K}}. \end{array}$$ (4)

In cSLO measurements, the retinal illuminance *I* is much bigger than $\frac{L}{K}$, and we evaluate *R* only at *t* much smaller than *K*. Therefore, Eq (4) reduces to $R(t)\approx e^{-\frac{I}{L}t},$ a simplification that was already pointed out in [5, 15].

### 2.3 Macular pigment model

To improve accuracy in rod rhodopsin mapping, we quantify macular pigments from multi-spectral autofluorescence image sets. In the past, we developed noninvasive multi-spectral autofluorescence imaging of the human retina by adding selected interference filter sets to standard fundus cameras, cf. [34, 37–43]. By exciting the fluorescent lipofuscin granules within the RPE, the Beer-Lambert model for the double path penetration enables us to effectively measure the spatial macular pigment distribution from a set of multi-spectral autofluorescence images. Quantitative measurements of macular pigments based on two-wavelength autofluorescence images have been introduced by Delori et al. in [33, 44]. To more robustly analyze the spatial macular pigment distribution, we introduce a multiple-wavelength model that enables more effective self-consistency tests.

Let *AF*_*f*_(Λ, *λ*) and *AF*_*p*_(Λ, *λ*) be the autofluorescence measured at the fovea and the perifovea, respectively. While *AF*_*f*_ depends on the specific location within the fovea, the term *AF*_*p*_ is often replaced by a circular average at 6 degrees [33]. We denote the optical density of the foveal and perifoveal tissue by *D*_*f*_ and *D*_*p*_, respectively. Let Φ_*f*_ and Φ_*p*_ be the fluorescence efficiencies of lipofuscin in the foveal and perifoveal regions. According to Eq (1), the foveal and perifoveal autofluorescence are given by

$$\begin{array}{ccc} {\text{AF}}_{f}\left(\Lambda ,\lambda \right) & = & I\left(\Lambda \right)\Phi_{f}\left(\Lambda ,\lambda \right)e^{-(D_{f}\left(\Lambda \right)+D_{f}\left(\lambda \right))},\\ {\text{AF}}_{p}\left(\Lambda ,\lambda \right) & = & I\left(\Lambda \right)\Phi_{p}\left(\Lambda ,\lambda \right)e^{-(D_{p}\left(\Lambda \right)+D_{p}\left(\lambda \right))}. \end{array}$$

We can assume that the fluorophore at the fovea has the same composition as that at the perifovea (constant shape of its spectrum over ${\left\{(\Lambda_{j},\lambda_{j})\right\}}_{j=1}^{n}$), and that foveal-perifoveal differences in absorption by other pigments (retinal blood, visual pigments, and RPE melanin) are negligible. We also neglect the low macular pigment concentration in the perifoveal region, so that the optical density of macular pigment *D*_*MP*_ at 460*nm* is the difference *D*_*MP*_(460*nm*) = *D*_*f*_(460*nm*) − *D*_*p*_(460*nm*), [33, 44]. We use the relative extinction coefficient *k*_*MP*_ of macular pigment, scaled to *k*_*MP*_(460*nm*) = 1, such that *D*_*MP*_(*λ*) = *k*_*MP*_(*λ*)*D*_*MP*_(460*nm*), and we obtain

$$\begin{array}{c} \log(\frac{{\text{AF}}_{p}\left(\Lambda ,\lambda \right)}{{\text{AF}}_{f}\left(\Lambda ,\lambda \right)})=\log(\frac{\Phi_{p}\left(\Lambda ,\lambda \right)}{\Phi_{f}\left(\Lambda ,\lambda \right)})+D_{MP}\left(460nm\right)\left(k_{MP}\left(\Lambda \right)+k_{MP}\left(\lambda \right)\right). \end{array}$$

We choose *n* pairs of excitation and emission wavelengths ${\left\{(\Lambda_{j},\lambda_{j})\right\}}_{j=1}^{n}$ and weights ${\left\{\omega_{j}\right\}}_{j=1}^{n}$ such that $\sum_{j=1}^{n}\omega_{j}=0.$ We apply the above equations for each wavelength pair (Λ_*j*_, *λ*_*j*_), multiply by *ω*_*j*_, and add them up to obtain

$$\begin{array}{c} \sum_{j=1}^{n}\omega_{j}\log(\frac{{\text{AF}}_{p}\left(\Lambda_{j},\lambda_{j}\right)}{{\text{AF}}_{f}\left(\Lambda_{j},\lambda_{j}\right)})=\sum_{j=1}^{n}\omega_{j}\log(\frac{\Phi_{p}\left(\Lambda_{j},\lambda_{j}\right)}{\Phi_{f}\left(\Lambda_{j},\lambda_{j}\right)})+D_{MP}\left(460nm\right)\sum_{j=1}^{n}\omega_{j}\left(k_{MP}\left(\Lambda_{j}\right)+k_{MP}\left(\lambda_{j}\right)\right). \end{array}$$

Since the foveal-perifoveal differences in fluorophore composition are negligible, the ratio $\frac{\Phi_{p}(\Lambda_{j},\lambda_{j})}{\Phi_{f}(\Lambda_{j},\lambda_{j})}$ does not depend on *j*. Therefore, we can determine *D*_*MP*_(460*nm*) by

$$\begin{array}{c} D_{MP}\left(460nm\right)=\frac{1}{\sum_{j=1}^{n}\omega_{j}\left(k_{MP}\left(\Lambda_{j}\right)+k_{MP}\left(\lambda_{j}\right)\right)}\log(\prod_{j=1}^{n}\frac{{\text{AF}}_{p}^{\omega_{j}}\left(\Lambda_{j},\lambda_{j}\right)}{{\text{AF}}_{f}^{\omega_{j}}\left(\Lambda_{j},\lambda_{j}\right)}). \end{array}$$ (5)

If we only choose two excitation wavelengths Λ_1_ = 480*nm* and Λ_2_ = 520*nm* with the weights 1 and −1, respectively, then we obtain the formula originally proposed in [33]. Our formula (5) enables several tests for self-consistency by removing or adding wavelength pairs and by changing the weights.

### 2.4 Combined model

To measure rod rhodopsin in the human retina, we first quantify the macular pigment density *D*_*MP*_(460) from multi-spectral autofluorescence image sets according to formula (5). The rod rhodopsin density can then be quantified from cSLO autofluorescence movies using the bleaching model. The macular pigment density is a parameter of the bleaching model whose exact specification increases the accuracy. By combining Eq (2) with the simplification of Eq (4), we derive the complete bleaching model

$$\begin{array}{c} \text{AF}\left(\Lambda ,\lambda ,t\right)=I\left(\Lambda \right)\Phi \left(\Lambda ,\lambda \right)e^{-(D_{MP}\left(460nm\right)\left(k_{MP}\left(\Lambda \right)+k_{MP}\left(\lambda \right)\right)+\varrho_{rh}\left(0\right)\left(k_{rh}\left(\Lambda \right)+k_{rh}\left(\lambda \right)\right)e^{-\frac{I}{L}t})}. \end{array}$$ (6)

Note that each flash of the fundus camera in macular pigment measurements fully bleaches rhodopsin so that our subsequent multi-spectral images are not affected by the bleaching kinetics and hence do not depend on the rod rhodopsin distribution.

### 2.5 Bleaching protocol

For recording rhodopsin bleaching cSLO movies, we follow a simple clinical protocol in which the human subject wears vermillion sunglasses (rod-protecting) while waiting and the photographer performs focus adjustment using the infrared reflection imaging (nonbleaching) in the cSLO. A 488*nm* excited autofluorescence movie (≈ 8 frames per sec, 1 minute long) is started that is recorded from the start with blinks every 10*s*, which refresh the tear film layer on the cornea. The average photon flux bleaches the rod rhodopsin after > 25*s*. We record the cSLO movie until steady-state rhodopsin bleaching.

## 3 Mathematical analysis of rhodopsin quantification

After clinical autofluorescence measurements are recorded supposedly satisfying Eq (6), we must solve for *ϱ*_*rh*_(0) at each pixel location. This requires advanced mathematical tools outlined in detail next.

### 3.1 Numerical rhodopsin extraction

The recorded cSLO movie yields the autofluorescence *AF*(Λ, *λ*, *t*) over time in Eq (6). For notational convenience, we shall denote the model parameters by

$$\begin{array}{c} \alpha =I\left(\Lambda \right)\Phi \left(\Lambda ,\lambda \right)e^{-D_{MP}\left(460nm\right)\left(k_{MP}\left(\Lambda \right)+k_{MP}\left(\lambda \right)\right)},\;\beta =\frac{I}{L},\;\gamma =\varrho_{rh}\left(0\right)\left(k_{rh}\left(\Lambda \right)+k_{rh}\left(\lambda \right)\right). \end{array}$$ (7)

The extinction coefficients *k*_*MP*_ and *k*_*rh*_ are derived from the literature. We find the rhodopsin density *ϱ*_*rh*_(0) by numerically determining the parameters *α*, *β*, and *γ* in

$$\begin{array}{c} \text{AF}\left(\Lambda ,\lambda ,t\right)=\alpha e^{-\gamma e^{-\beta t}},\;\text{for}\;t\geq T_{0}\text{,} \end{array}$$ (8)

where *T*_0_ ≈ 0 is the time of initial exposure to light. Note that we have bleaching curves *f*(*t*), *t* ∈ [*T*_0_, *T*], associated to *AF*(Λ, *λ*, *t*), for each pixel location (*x*, *y*). Computed parameters are optimized within the declared model, but may differ from the actual physiological parameters *α*, *β*, *γ* by a small error margin, or differ in certain areas due to physiological phenomena that are not present in the model. So, to avoid confusion, we will denote the numerically recovered parameters by $\hat{a}(x,y)\approx \alpha (x,y)$, $\hat{b}(x,y)\approx \beta (x,y)$, and $\hat{c}(x,y)\approx \gamma (x,y)$.

Given a set of autofluorescence measurements *f*(*t*) satisfying the model for *AF*(Λ, *λ*, *t*), the numerical scheme approximates the bleaching process in the form of the double-exponential law in Eq (8). To avoid the use of double-exponents, we consider a “mean-square-log” deviation between *f*(*t*) and *AF*(Λ, *λ*, *t*),

$$\begin{array}{c} E\left(a,b,c\right)=\int {\left|\ln(f(t))-\ln(\text{AF}(\Lambda ,\lambda ,t))\right|}^{2}dt=\int {\left|\ln\left(f\left(t\right)\right)-\ln\left(a\right)+ce^{-bt}\right|}^{2}dt, \end{array}$$ (9)

and numerically determine the three model parameters $\hat{a}$, $\hat{b}$, and $\hat{c}$ that minimize *E*. We want to point out that this energy is different from the one used in our earlier preliminary work [38, 39], where we directly enforced *f*(*t*) ≈ *ae* ^−*ce*^−*bt*^^. In the present approach Eq (9), we would like $\frac{f(t)}{ae^{-ce^{-bt}}}$ to be close to 1, which implies that $\text{ln}(\frac{f(t)}{ae^{-ce^{-bt}}})=\text{ln}(f(t))-\text{ln}a+ce^{-bt}$ should be close to 0.

**Remark 3.1** The remainder of the mathematical section is dedicated to develop and validate an iterative algorithm that determines the global minimizer of *E* in Eq (9). We shall see that the Hessian matrix of *E* is positive definite throughout, so that zeros of the first partial derivatives not only yield local minimizers but indeed the global minimizer. Therefore, the iterative scheme will indeed converge towards the global minimizer of Eq (9).

### 3.2 Implementation details

To find the actual minimizers of *E*, we set *p* = ln(*a*) and use a gradient descent algorithm. The latter algorithm can equivalently be derived from solving the Euler-Lagrange equations associated to Eq (9),

$$\begin{array}{c} 0=\partial_{p}E,\;0=\partial_{b}E,\;0=\partial_{c}E, \end{array}$$ (10)

with the use of an artificial time marching [45]. Indeed, the gradient descent of *E* can be formulated as a discretization of the following system

$$\begin{array}{c} \partial_{\tau }p=-\partial_{p}E,\;b_{\tau }=-\partial_{b}E,\;c_{\tau }=-\partial_{c}E, \end{array}$$ (11)

where *a*, *b* and *c* are now supposed to be functions depending on some artificial time parameter *τ*. More precisely, the steady-states of Eq (11) satisfy Eq (10) and yield the minimizer of *E* in Eq (9), so that we can set

$$\begin{array}{c} \hat{a}=\lim_{\tau \to \infty }e^{p(\tau )},\;\hat{b}=\lim_{\tau \to \infty }b\left(\tau \right),\;\hat{c}=\lim_{\tau \to \infty }c\left(\tau \right), \end{array}$$ (12)

cf. [45]. To actually compute the steady-states lim_*τ* → ∞_ *a*(*τ*), lim_*τ* → ∞_ *b*(*τ*), and lim_*τ* → ∞_ *c*(*τ*), we use a so-called semi-explicit finite difference scheme [46] that leads to

$$\begin{array}{ccc} \frac{p_{n+1}-p_{n}}{\tau_{p}} & = & 2\int_{T_{0}}^{T}\left(\ln\left(f\left(t\right)\right)-p_{n+1}+c_{n}e^{-b_{n}t}\right)dt,\\ \frac{b_{n+1}-b_{n}}{\tau_{b}} & = & 2\int_{T_{0}}^{T}\left(\ln\left(f\left(t\right)\right)-p_{n}+c_{n}e^{-b_{n}t}\right)c_{n}te^{-b_{n}t}dt,\\ \frac{c_{n+1}-c_{n}}{\tau_{c}} & = & -2\int_{T_{0}}^{T}\left(\ln\left(f\left(t\right)\right)-p_{n}+c_{n+1}e^{-b_{n}t}\right)e^{-b_{n}t}dt, \end{array}$$

where *τ*_*p*_, *τ*_*b*_, and *τ*_*c*_ are the individual step widths and initial values *p*_0_, *b*_0_, and *c*_0_ that still need to be determined. As usual, the integral shall be replaced with the finite sum over the measured time points *t*_1_, …, *t*_*m*_, i.e., only ${\left\{f(t_{i})\right\}}_{i=1}^{m}$ are available, and the index *n*+1 indicates the updated value of the solution at the next step, the index *n* corresponds to the “current” parameter values that were updated at the previous step. The second equation is explicit, i.e., we use *b*_*n*_ on the right-hand side. The first and third equations can be implicit, i.e, the index *n*+1 is used on the right-hand side, because we are still able to solve the equations by

$$\begin{array}{c} p_{n+1}=\frac{p_{n}+2\tau_{p}\int_{T_{0}}^{T}\left(\ln\left(f\left(t\right)\right)+c_{n}e^{-b_{n}t}\right)dt}{\left(1+2\tau_{p}\left(T-T_{0}\right)\right)}, \end{array}$$ (13)

$$\begin{array}{c} b_{n+1}=b_{n}+2\tau_{b}c_{n}\int_{T_{0}}^{T}\left(\ln\left(f\left(t\right)\right)-p_{n}+c_{n}e^{-b_{n}t}\right)te^{-b_{n}t}dt, \end{array}$$ (14)

$$\begin{array}{c} c_{n+1}=\frac{c_{n}-2\tau_{c}\int_{T_{0}}^{T}\left(\ln\left(f\left(t\right)\right)-p_{n}\right)e^{-b_{n}t}dt}{\left(1+2\tau_{c}\int_{T_{0}}^{T}e^{-2b_{n}t}dt\right)}. \end{array}$$ (15)

According to Eq (12), the minimizers of Eq (9) are computed from the iterations (13), (14), (15) as

$$\begin{array}{c} \hat{a}:=\lim_{n\to \infty }e^{p_{n}},\;\hat{b}:=\lim_{n\to \infty }b_{n},\;\hat{c}:=\lim_{n\to \infty }c_{n}. \end{array}$$ (16)

We shall verify the legitimacy of the above iterative scheme in Section 3.3.

Before, we still need to discuss the choice of the initial values *p*_0_, *b*_0_, and *c*_0_ for the respective model parameters. To determine their order of magnitude, we utilize a spatially averaged bleaching curve, so that the fitting is numerically stable, and we then use the resulting steady state solutions as initial values for consequent pixelwise processing. The fitting routine is, in fact, applied to the modified image stack, in which each pixel is averaged over its surrounding 8 × 8 pixels. The latter effectively reduces noise, including local distortions due to the eye movements and the mutual image registration.

We also see the importance to determine the macular pigment density a-priori because it influences the spatial distribution of the initial value *p*_0_ = ln(*a*_0_) that corresponds to *α* in Eq (7). In other words, the macular pigment measurements enable us to use more spatially accurate initial values of *p*_0_ yielding faster convergence of the numerical algorithms, so that overall accuracy is improved.

From the biophysical point of view it appears reasonable to assume that *β*, being the quotient between the steady-light illuminance *I* and the bleaching constant *L*, is constant throughout the image. Thus, we only minimize over *p* and *c*, and we shall support this simplification in Section 6.1.

### 3.3 Mathematical validation of the energy minimization

If we restrict the values of parameters to a closed bounded set of the form *a* ∈ [0, *A*], *b* ∈ [0, *B*], *c* ∈ [0, *C*], then *E*(*a*, *b*, *c*) in Eq (9) attains its nonnegative minimum. Since we keep the parameter *b* fixed, we only consider the first and third equations in Eq (11), which are

$$\begin{array}{c} \partial_{\tau }p=-2\int_{T_{0}}^{T}\left(\ln\left(f\left(t\right)\right)-p+ce^{-bt}\right)dt,\;\partial_{\tau }c=2\int_{T_{0}}^{T}\left(\ln\left(f\left(t\right)\right)-p+ce^{-bt}\right)e^{-bt}dt. \end{array}$$ (17)

According to a generalization of the Cauchy-Picard-Lindelöf Theorem any initial condition *p*(0) = *p*_0_ and *c*(0) = *c*_0_ yields a unique solution of this system of ordinary differential equations for all time provided that all second order partial derivatives of the right hand sides are uniformly bounded. Indeed, the second order partial derivatives in this case are

$$\begin{array}{c} |\partial_{p}^{2}E|=2\int_{T_{0}}^{T}1dt=2\left(T-T_{0}\right), \end{array}$$ (18)

$$\begin{array}{c} |\partial_{c}^{2}E|=2\int_{T_{0}}^{T}e^{-2bt}dt\leq 2\left(T-T_{0}\right), \end{array}$$ (19)

$$\begin{array}{c} |\partial_{p}\partial_{c}E|=2\int_{T_{0}}^{T}e^{-bt}dt\leq 2\left(T-T_{0}\right), \end{array}$$ (20)

so that the boundedness condition holds. Thus, the system (17) has solutions *p*(*τ*) and *c*(*τ*) defined for all artificial time *τ*. We now would like to claim that they converge towards a steady-state as the artificial time *τ* tends to infinity, and that $\hat{a}:={\text{lim}}_{\tau \to \infty }e^{p(\tau )}$ and $\hat{c}:={\text{lim}}_{\tau \to \infty }c(\tau )$, consistently with Eq (12), yield the minimizer of *E*.

To be precise, if we fix *b*, then Eq (17) are necessary conditions a minimizer of *E* must satisfy. To show that the parameters found by solving the system (17) indeed yield the minimum of *E*, we need to check that the Hessian matrix of *E* is positive definite. The latter, together with Eq (17), is a sufficient condition and equivalent to

$$\begin{array}{c} \partial_{p}^{2}E>0,\;\det(\begin{array}{cc} \partial_{p}^{2}E & \partial_{p}\partial_{c}E\\ \partial_{c}\partial_{p}E & \partial_{c}^{2}E \end{array})>0. \end{array}$$

Since $\partial_{p}^{2}E=2(T-T_{0})>0$ is always satisfied, we only need to check on the determinant. Its definition and the Cauchy-Schwartz inequality applied to the two functions *e* ^−*bt*^ and 1 yield

$$\begin{array}{ccc} \det(\begin{array}{cc} \partial_{p}^{2}E & \partial_{p}\partial_{c}E\\ \partial_{c}\partial_{p}E & \partial_{c}^{2}E \end{array}) & = & 4\left(T-T_{0}\right)\int_{T_{0}}^{T}e^{-2bt}dt-4{\left(\int_{T_{0}}^{T}e^{-bt}dt\right)}^{2}\\ & \geq & 4\left(T-T_{0}\right)\int_{T_{0}}^{T}e^{-2bt}dt-4\int_{T_{0}}^{T}e^{-2bt}dt\int_{T_{0}}^{T}1dt\\ & \geq & 4\left(T-T_{0}\right)\int_{T_{0}}^{T}e^{-2bt}dt-4\int_{T_{0}}^{T}e^{-2bt}dt\left(T-T_{0}\right)=0. \end{array}$$

The inequality in these computations becomes an equality only if the two functions *e* ^−*bt*^ and 1 are linearly dependent, which would require *b* = 0. We fix *b* > 0 anyway, so that the Hessian of *E* is positive definite. Therefore, there is only one critical point of the energy *E* in Eq (9), which must be the global minimum. According to Eq (16), the gradient descent (13) and (15) yields this minimizer as *n* tends to infinity.

## 4 Postprocessing: inpainting retinal blood vessels

Blood in visible retinal vessels is a dominating absorber of excitation wavelengths used to excite RPE autofluorescence, so that pixels containing these blood vessels do not detect lipofuscin from the underlying RPE. The detected fluorescence signal in these pixels represents largely dark noise, which does not exhibit the bleaching behavior described by the model in Section 2.4. Further inaccuracies in pixels at retinal vessel edges are caused by the micro-movements of the eye during recording and image registration imprecision.

As postprocessing, we shall remove retinal blood vessels from the rhodopsin map requiring retinal vessel detection and sophisticated mathematical techniques for image inpainting. The latter means that we mask the blood vessels in the rhodopsin maps and treat those pixels as occlusions (or missing pixels) in the context of an inpainting problem, in which surrounding pixels “diffuse” into occlusion areas. We shall recover the unknown intensity values in missing areas by using a technique that was shown to be efficient in restoring a wide variety of natural images [47].

### 4.1 Detecting blood vessels

Retinal blood vessel detection has already been widely addressed in the literature [48–56], and, in principle, we could choose one of the standard tools, see also [57, 58] for related approaches. Nevertheless, we shall describe two semi-automated methods producing a mask of the pixels to be inpainted that directly evolves from the rhodopsin model itself.

1. Let *f*(*x*, *y*, *t*) denote our image stack, where (*x*, *y*) keeps track of the pixel location. For each fixed pair (*x*, *y*), the numerical minimization algorithm provides the outputs $\hat{a}(x,y)$, $\hat{b}(x,y)$, $\hat{c}(x,y)$, as well as the number of iterations *N*(*x*, *y*) required for the gradient descent to converge. The “image” or “matrix” *N* contains edge information that enables tracing contours including the edges of the blood vessels. Thus, the speed of convergence of the numerical minimization scheme provides a tool to detect the retinal blood vessels.
2. The second method relies on the error between measured intensities and reconstructed autofluorescence from the computed model parameters. In other words, we aim to detect the pixel locations (*x*, *y*) where the cSLO measurements *f*(*x*, *y*, *t*) strongly deviate from the proposed bleaching model Eq (8). For instance, we can check the magnitude of $E(\hat{a},\hat{b},\hat{c})$. Here, instead of the squared logarithmic deviation in $E(\hat{a},\hat{b},\hat{c})$, we use the total *L*_1_ deviation at each pixel location, i.e,
  $$\begin{array}{c} \Delta \text{AF}\left(x,y\right)=\sum_{i=1}^{m}\left|f\left(x,y,t_{i}\right)-\hat{a}\left(x,y\right)e^{-\hat{c}\left(x,y\right)e^{-\hat{b}\left(x,y\right)t_{i}}}\right|, \end{array}$$
  where *t*_1_, …, *t*_*m*_ denote the time points associated to the frames in the recorded cSLO movie. Thresholding of the values between varying percentiles enables the identification of pixels, where the model does not fit the actual measurements, hence detecting the blood vessels. We denote those pixel locations by Ω.

### 4.2 Inpainting technique

Image inpainting is an active mathematical research field and several techniques have been proposed in the literature [59–69]. Here, we need image inpainting to remove the blood vessels as occlusions from the rhodopsin distribution map. Due to its robustness against measurement noise, we shall use a refined variational method developed in [37, 47] for binary images, briefly described in the following. Since the cSLO camera output is 8-bit grayscale, we use bit-wise processing (work with 8 binary images separately) as outlined in [47], which further suppresses distortions effectively. Mathematically, we identify the images with binary functions *g*:[0, 1]^2^\Ω → {0,1}, and Ω ⊂ [0, 1]^2^ is the location of the retinal blood vessels to be inpainted, so that we are looking for values of *g* on Ω. We compute the desired function $\hat{u}:{\left[0,1\right]}^{2}\to \mathbb{R}$ as a minimizer of the modified (wavelet) Ginzburg-Landau energy,

$$\begin{array}{c} E_{\text{WGL}}\left(u\right)=\int_{{\left[0,1\right]}^{2}\setminus \Omega }{\left|u(x,y)-g(x,y)\right|}^{2}d\left(x,y\right)+\frac{1}{2\epsilon \mu }\int_{{\left[0,1\right]}^{2}}u^{2}\left(x,y\right){\left(u(x,y)-1\right)}^{2}d\left(x,y\right)+\frac{\epsilon }{\mu }{\left|u\right|}_{B}^{2}, \end{array}$$ (21)

and refer to [47] for the detailed motivation, see also [65]. Briefly, the first term forces the minimizer $\hat{u}$ to be close to *g* on [0, 1]^2^\Ω, so that only minimal changes in intensities outside blood vessels are allowed. The second and third term refer to the underlying Ginzburg-Landau model for *u*, meaning regularity conditions on *u* and the way *g* can be extended to Ω. For the reader familiar with such techniques, the term ${|u|}_{B}^{2}$ denotes the Besov 1-2-2 norm computed using a wavelet expansion of *u*, and the details are described in [47]. Such variational methods based on optimization commonly yield much better results than more elementary interpolation techniques.

The pixels of retinal vessels and hence the missing area Ω are not perfectly well-defined due to measurement inaccuracies or micro-movements of the eye during the recordings and resulting image registration artifacts. To compensate for this, we replace the first term in Eq (21) with

$$\begin{array}{c} \int_{{\left[0,1\right]}^{2}}\chi_{\Omega }\left(x,y\right){\left|u(x,y)-g(x,y)\right|}^{2}d\left(x,y\right), \end{array}$$ (22)

where the mask *χ*_Ω_ : [0, 1]^2^ → [0, 1] smoothly changes from 0 to 1 at the “boundary” of Ω in [0, 1]^2^, thus gradually assigning a lower weight to the forcing *u*(*x*, *y*) ≈ *g*(*x*, *y*) close to the unknown area. The final rhodopsin map then is a result of a two-step procedure. At the first step, three versions of the map were recovered using inpainting with slightly varying parameters of *ϵ*, *μ* in Eq (21) and smoothening of the mask *χ*_Ω_ in Eq (22)—meaning the information near the vessels was given different levels of priority each time. At the second step, the average of the resulting maps was subject to adaptive binary wavelet thresholding, which eliminates further pixel artifacts, see [47] for details about the technique.

## 5 Summary of the numerical scheme

We first compute the macular pigment map. Second, we use the knowledge of the spatial macular pigment distribution to specify the initial values of the numerical fitting scheme when computing the rhodopsin map. Third, we detect retinal blood vessels to remove them from such maps and use image inpainting as outlined in the previous section yielding the final rod rhodopsin maps.

## 6 Results

We derived the methodology to quantify the rod rhodopsin distribution from cSLO movies that show autofluorescence brightening to a steady-state level as the rhodopsin bleaches. The present results section addresses three points. First, we shall compute the spatial variation of $\hat{b}$ when minimizing *E* over all three parameters *a*, *b*, *c*, motivating the paradigm to keep *b* spatially constant. Second, we show the spatial rhodopsin distribution maps derived from numerical optimization, vessel detection and inpainting. Third, the characteristics of the computed rhodopsin maps are compared to the rod distributions derived in [7–9].

### 6.1 Numerical algorithm

**Fig 1:**
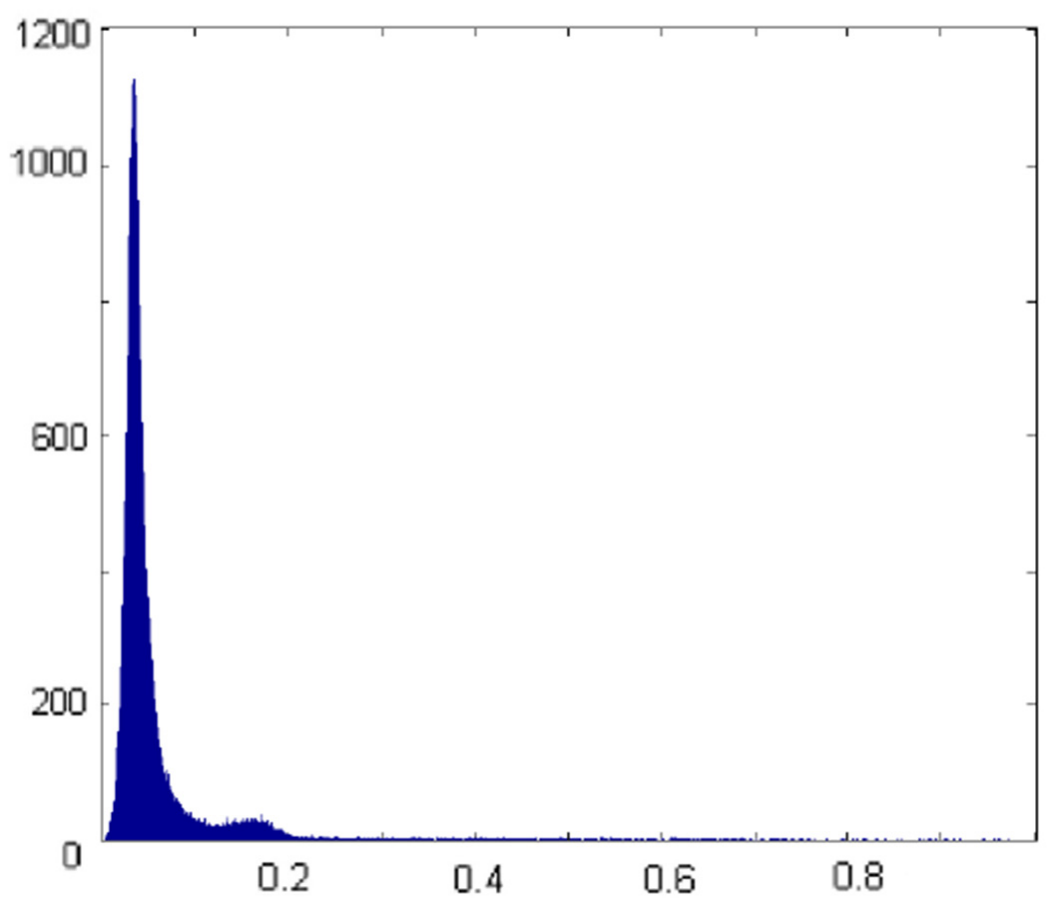
Histogram of computed parameter *b*. We optimized all 3 parameters *p*, *b*, and *c* at the same time and plotted a representative histogram of parameter *b*. The counts cluster tightly around the mean, enabling us to assign the highest count to *b*_0_, keep it fixed throughout the image and only optimize over the remaining two parameters *p* and *c*.

The parameter *β* is a fundamental biophysical constant of rhodopsin-photon absorption and thus is presumed to be spatially invariant in our analysis, so that we only optimize over *p* and *c* [18, 35]. We shall validate this approach by observing that the optimization over all 3 parameters leads to similar results. It should be mentioned that computed parameters $\hat{a}$, $\hat{b}$, and $\hat{c}$ turn out positive without explicit enforcement. When solving Eq (10) for all 3 parameters by applying Eqs (13), (14), and (15), we see only minor variations of *b*(*x*, *y*) away from the blood vessels, the fovea and the optic disc. More precisely, we computed an average of the intensity curves over a large annulus-like area (5–8 degrees) away from the fovea and ran the fitting routine for the averaged experimental curve. A representative histogram of the recovered values of *b*, cf. Fig 1, shows strong concentration around one value, which we can then assign to *b*_0_.

### 6.2 Inpainted rhodopsin maps

**Fig 2:**
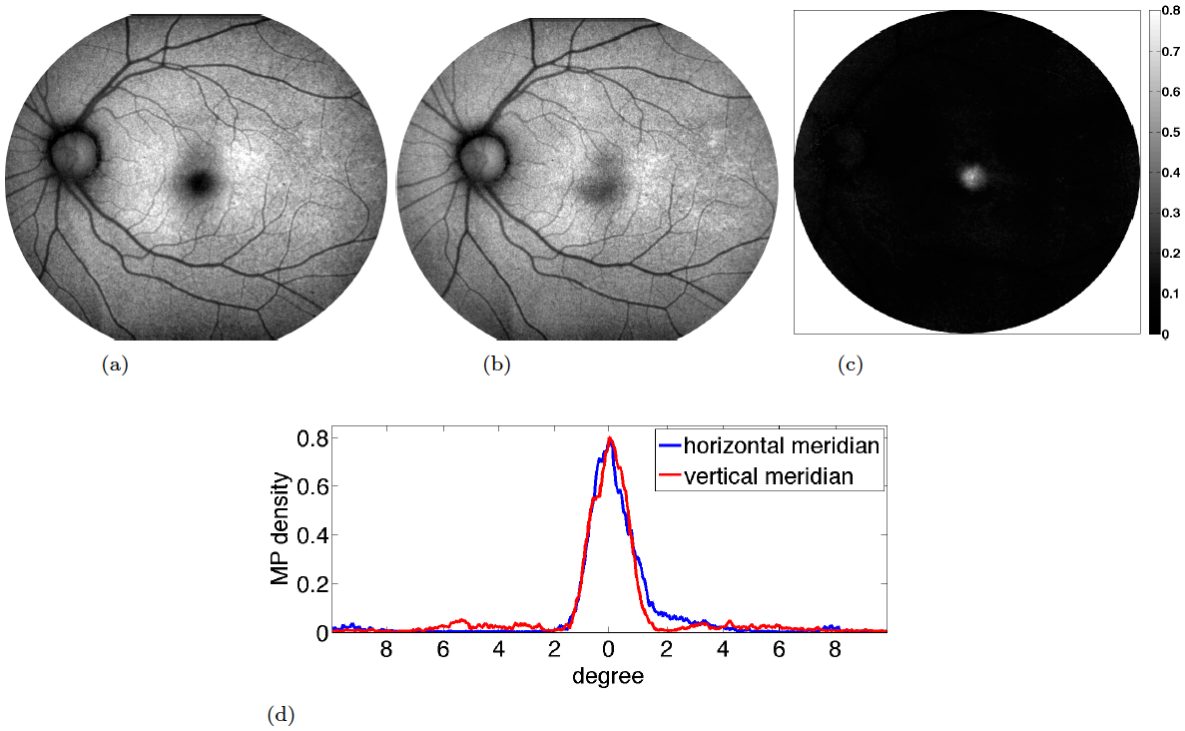
Macular pigment measurement. Two bands of a multi-spectral autofluorescence image set are shown and a representative macular pigment map derived from formula (5), where we optimized the map over several choices of weights to maximize self-consistency of the measurement. The macular pigment is concentrated in the macula and rapidly decays with distance to the center of the fovea. Although vessels and optic disc can still be recognized visually, the pixel magnitudes are so small that their contribution in the further steps is negligible. (a) blue excitation (b) yellow excitation (c) spatial map of MP density (d) horizontal and vertical MP profiles, averaged over small stripes.

**Fig 3:**
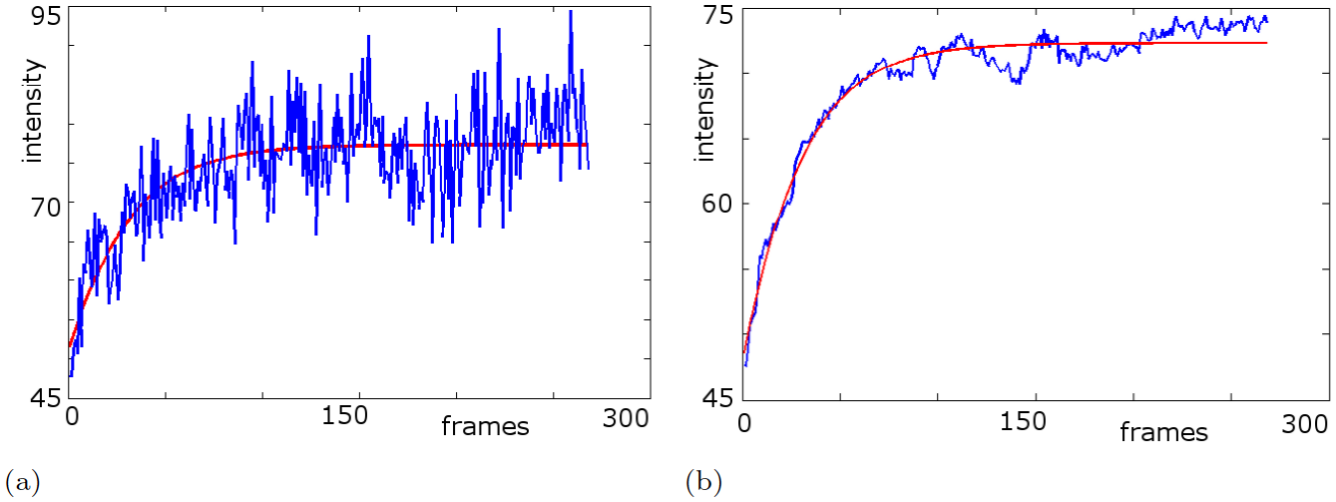
Bleaching curve and its fit (I). Temporal sequences of the intensity values (blue) and the corresponding fit (red) through minimizing *E* in Eq (9). Due to the low signal to noise ratio in cSLO measurements, there are large variations, but we can still recognize the overall trend. By averaging the cSLO movie over an angulus of 3 degree width, the noise and hence the variation are suppressed and the fit becomes tighter. (a) typical temporal sequence with its fitted curve at one pixel (b) temporal sequence from averaging intensities over an annulus (5–8 degrees) and fitted curve.

**Fig 4:**
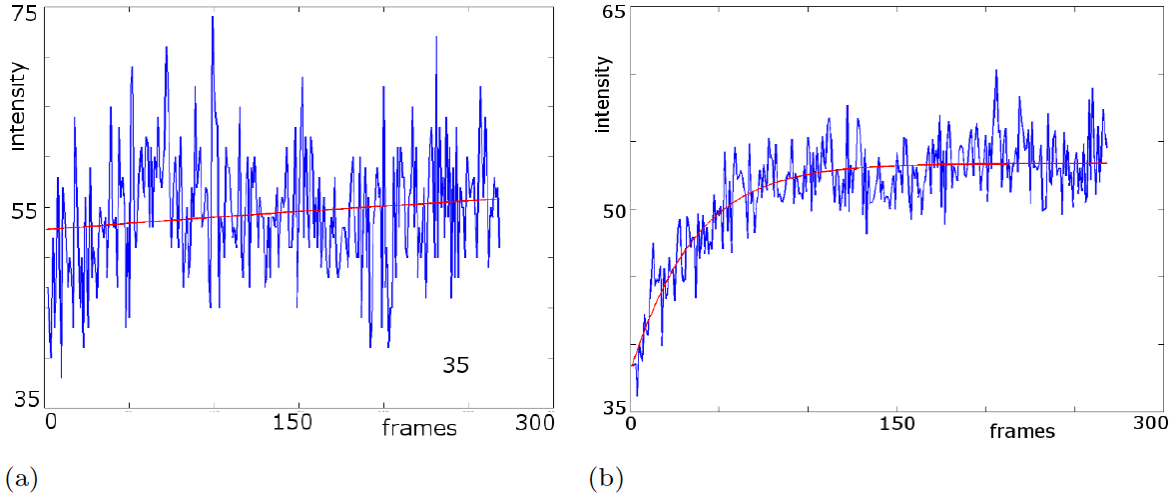
Bleaching curve and its fit (II). The fit near the fovea does not show the typical bleaching behavior because there are almost no rods present. Instead, the minimization procedure leads to a linear fit. By averaging an 8 by 8 pixel region outside the fovea, we derive a typical bleaching curve with a good fit. All further computations are computed using this 64 pixel averaging to effectively suppress the noise in the cSLO measurements. (a) non-averaged image stack, curve chosen near the fovea, parameters (b) averaged over square neighborhoods (8 × 8 pixels).

**Fig 5:**
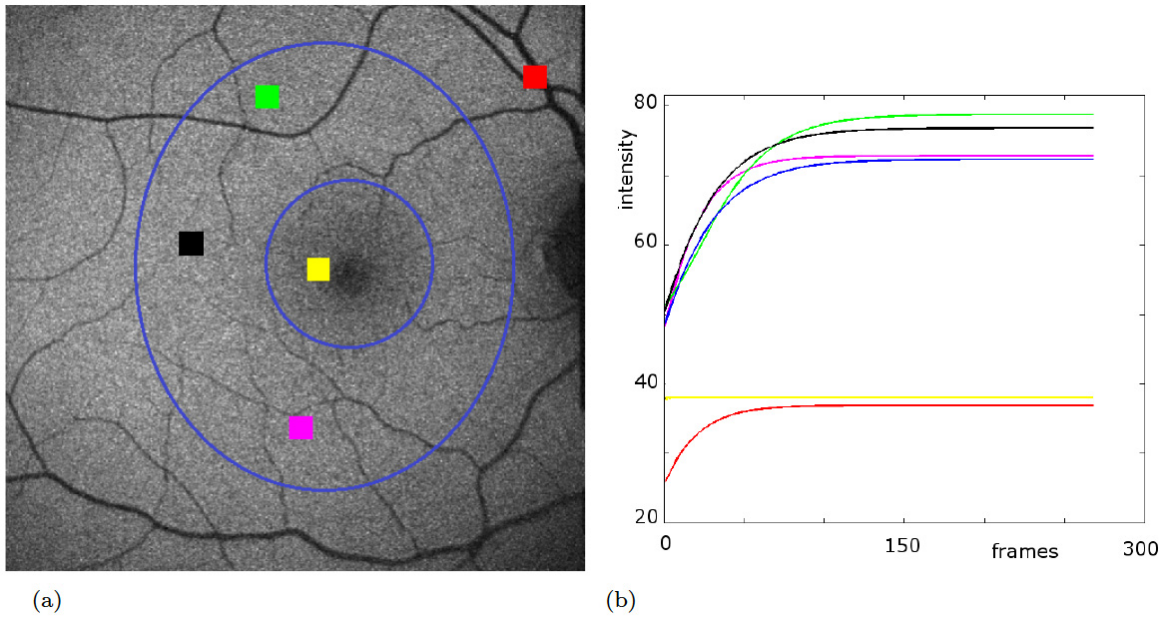
Local fitted curves. Bleaching curves computed from fitted parameters averaged over several small spatial regions. The blue curve is the fitting for the intensity vector averaged over the annulus-like area between two blue ovals shown in (a). Green, black, and purple curves show similar bleaching kinetics. The yellow curve corresponds to the region close to the fovea where not much rhodopsin is expected and the fit becomes a steady line. Blood vessels do not exhibit the bleaching model although the fit (red) still shows some bleaching behavior. Nevertheless, the intensity values appear far off. (a) regions marked on the first image of the stack (b) respective fitted bleaching curves computed using the vectors of the averaged intensities.

**Fig 6:**
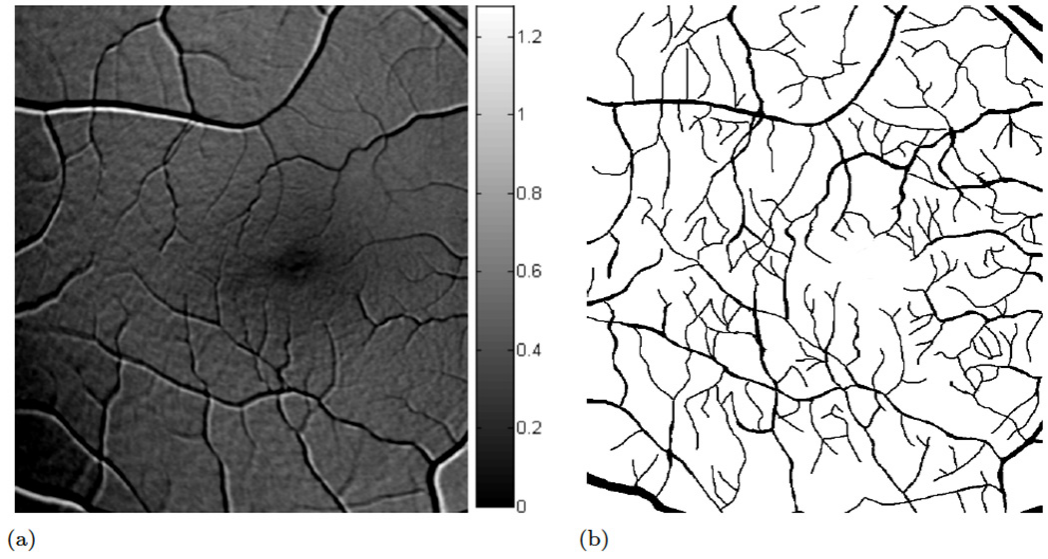
Rod rhodopsin measurement (I) and Blood vessel detection. We determined *b* ≈ 0.04 through the largest count in the histogram, which corresponds to the fit associated to an average of a large annulus area. The gradient descent minimization scheme led to the optimized parameters $\hat{a}$ and $\hat{c}$, where the macular pigment distribution was used to refine the initial value *a*_0_. Blood vessels show up as artifacts in the resulting rhodopsin maps and must still be removed. We detect blood vessels through a scheme that evolves from the computational minimization of *E* related to the rhodopsin model. Spatial regions where the numerical algorithm converges significantly slower than in other image parts shall be identified as blood vessels. The number of required iterations leads to an image which can simply be thresholded to identify retinal blood vessels. (a) spatial map of parameter $\hat{c}$ optimal within the model. Brighter means increase in rhodopsin density, but brightest spots occur as registration artifacts along blood vessels. Therefore, the grayscale colormap is suppressed. (b) extracted mask of blood vessels derived from thresholding the number of iterations needed for convergence in the optimization scheme. Simple pixel growth in the mask would lead to complete coverage of the vessels.

**Fig 7:**
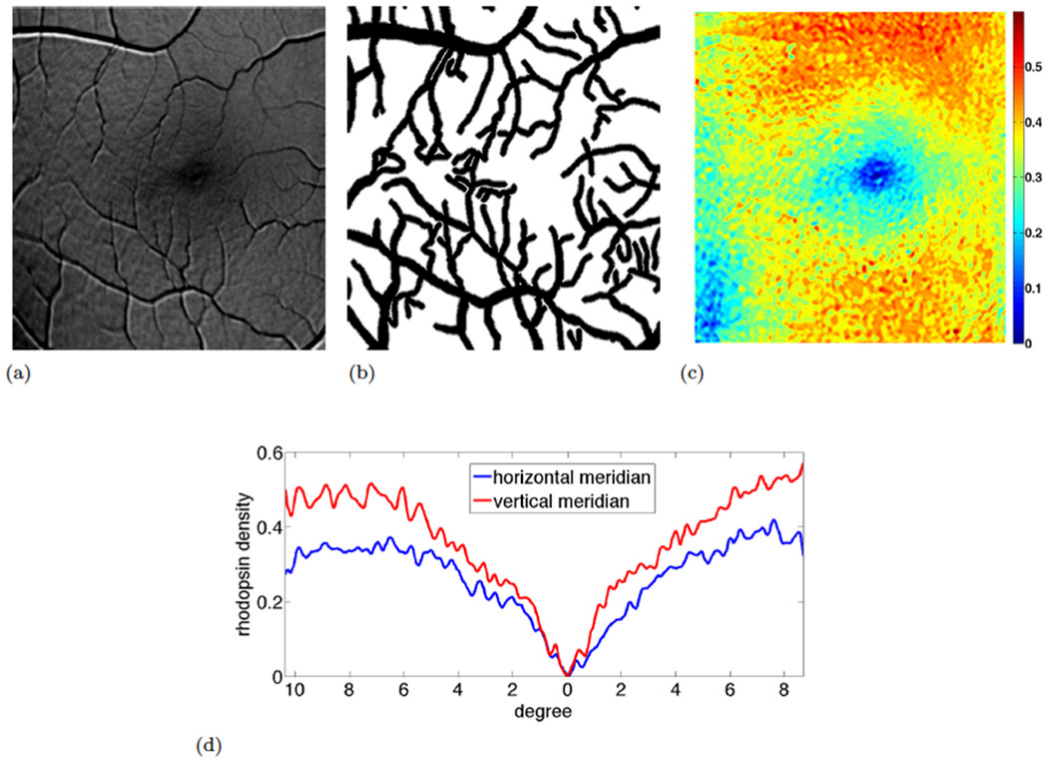
Rod rhodopsin measurement (II). To derive the final rhodopsin map, we first detect the blood vessels. Instead of a binary vessel mask, we use a gradual interface at vessel borders resulting in a smooth vessel mask as proposed in Eq (22). Inpainting based on Eq (21) was used to remove the retinal blood vessels from the final rhodopsin map leading to a smooth rod rhodopsin map. We show the computed parameter *γ*, which is the sum of emission and excitation rhodopsin absorbance. Since the emission rhodopsin absorbance (590*nm*-600*nm*) is neglible, *γ* indeed is the rhodopsin absorbance at 488*nm*. The center of the fovea lacks rhodopsin whose density increases when moving apart from the center. Consistently with the rod distribution described in [8], rod rhodopsin increases most rapidly along the superior vertical meridian and increases least rapidly along the nasal horizontal meridian. Although we do not see a connected hot spot of highest rod rhodopsin density, we observe larger and more connected areas of highest rhodopsin density in the superior retina than in the inferior retina, again, consistent with the rod distribution in [8]. (a) cropped rhodopsin map from Fig 6a to be inpainted (b) smooth mask *χ* used in the spatial consistency forcing term (c) the final output showing the distribution of the rhodopsin optical density *γ*, i.e., the rhodopsin absorbance at 488*nm*. Units are suppressed to indicate that we compute its distribution rather than absolute quantitative optical densities as we are not able to validate overall amplitudes due to background in our image sets. (d) horizontal and vertical rhodopsin profiles, averaged over small stripes.

The main objective of the present methodology paper is to develop the entire cycle of rod rhodopsin quantification, ranging from the biophysical model, the clinical measurements, and all the way to the mathematical optimization procedures and image analysis tools. Indeed, after retinal images are acquired and registered, building the rod rhodopsin maps from cSLO measurements is a 5-step procedure:

1. macular pigment:
  We spatially quantified macular pigment from multi-spectral autofluorescence image sets refined through self-consistency tests in Fig 2.
2. initial values:
  While Fig 3a shows a typical bleaching curve of a single pixel, we compute initial values *a*_0_, *b*_0_, and *c*_0_ from averages over a large spatial area in the autofluorescence movie, cf. Fig 3b. The parameter *b*_0_ is determined as the maximal count in the underlying histogram as described in Section 6.1, and *a*_0_ is spatially modified according to the macular pigment maps.
3. preliminary rhodopsin map:
  We then apply the gradient descent to perform the minimization on 8 × 8 pixel patches to reduce noise. Parameters for pixels near the fovea or near the major blood vessels show atypical behavior and the fitting changes qualitatively, Figs 4, 5. When ignoring such areas, plotting the parameter $\hat{c}$ yields a preliminary rhodopsin map in Fig 6a.
4. vessel detection:
  We detect retinal blood vessels to remove them from the preliminary rhodopsin maps, see Fig 6b.
5. inpainting:
  We inpaint the blood vessels to obtain the final rod rhodopsin map in Fig 7. The deviation between the preliminary rhodopsin map and those after image inpainting, assessed away from the mask, are below 5% in the amplitude, and < 1% in the mean-square error.

### 6.3 Rhodopsin maps compared to rod photoreceptor topology

We compare our rhodopsin distribution maps with typical rod topology as characterized in [7–9]. Note that we compare the distribution meaning comparison of actual densities in a qualitative but not quite quantitative fashion. Thus, local rhodopsin changes can be observed but the overall amplitude of our maps is arbitrary to some extent, so that we suppress units in our figures. There are no rods nor rhodopsin present in the fovea, and rhodopsin density increases with distance to the foveal center, Fig 7c. As observed in rod photoreceptor cells [8], the rhodopsin density increases most rapidly along the superior vertical meridian and less rapidly along the nazal horizontal meridian, see Fig 7d.

## 7 Discussion

Our analysis of cSLO lipofuscin AF image sequences in modestly (rod-protecting sun glasses and dim room lights) dark-adapted subjects is based on a physiological model of rhodopsin bleaching kinetics [18] to provide a rapid means for spatially mapping of the rhodopsin distribution within rod photoreceptors over a 30 degree field of view. Our procedure for mathematical optimization of these maps was guided by an interdisciplinary integration of the clinical image acquisition process, the biophysical models, and the mathematical analysis together with the image processing. The numerical scheme is modular, so that our methods for parameter fitting, vessel detection and image inpainting could be replaced with other tools, more familiar to any given clinical team. Nevertheless, our proposed methods use state-of-the-art in applied mathematics to provide improved numerical stability and reproducibility. Both models for macular pigment absorption and rhodopsin bleaching with regeneration used in our analysis, have been validated in the literature. Recently Morgan and Pugh [27] have published a method for measuring time-dependent rhodopsin bleaching based on reflected light using a cSLO device. Their method requires spatial averaging over specifically illuminated 4.8-degree retinal fields, which represents an additional clinical processing step. Our current method and analysis is advantageous in that it obtains reliable maps of bleachable rhodopsin and RPE autofluorescence levels simultaneously at relatively high (≈ 50*μm*) resolution for a large (30 degree) retinal field from a brief (1 minute) clinical imaging sequence.

Melanin granules within the RPE undergo changes over the human lifespan, increasingly being converted by lysomal processing into lipofuscin-coated melanolipofuscin granules. They are uniformly dispersed among lipofuscin granules compared to the more apical distribution of RPE melanin observed at earlier ages, cf. [32, 70]. Such changes increase the local excitation light levels reaching fluorescent bisretinoids. Where melanin becomes substantially oxidized its visible absorption is reduced leading to increases in detected local autofluorescence. However, the effect of melanin changes with age or pathology should not affect the local rod irradiance and therefore neither the local kinetics nor fractional amplitude seen in our analysis of the cSLO AF imaging sequences. Thus, it seems reasonable to explicitly model the macular pigment signal attenuation but neglect the melanin contribution. The physiological variables *α*(*x*, *y*), *β*(*x*, *y*), and *γ*(*x*, *y*) appear to be consistent as we optimize the model parameters using the steady state of a system of ODEs. Our model and parameter optimization provide plausible maps of rod rhodopsin distributions in normal individuals using a clinically simple 1*min* noninvasive imaging sequence. The fitting curves enable us to detect local rhodopsin changes up to a resolution of 50*μm*, which is competitive to other standard clinical instrument, and higher resolution is usually only achieved by more specialized instruments. Our rod rhodopsin maps are consistent with characteristics of typical rod distribution observed in the literature [7, 9], which appears to qualitatively validate our approach. Rod density increases most rapidly along the superior vertical meridian and increases least rapidly along the nasal horizontal meridian, just as in our rod rhodopsin map. We observe larger and more connected areas of highest rhodopsin density in the superior retina than in the inferior retina, again, consistent with the rod distribution in [8].

We must point out though that we claim spatial mapping of rhodopsin distribution that provides qualitative spatial rhodopsin density maps. Image background in standard fundus and cSLO cameras prevents us at this point in time from claiming quantitative spatial mapping of the actual rod rhodopsin density. Image background needs to be removed before our actual numerical scheme becomes active leading to amplitude differences that depend on the method and amount of background removal. Nonetheless, our claim of enabling detection of spatial variations in rod rhodopsin density appears valid and may be sufficient to identify clinically relevant early pathological changes. Once background removal is solved satisfactorily as an additional module in our image analysis pipeline, our proposed method would immediately enable spacial mapping of rod rhodopsin density in a quantitative fashion.

Given the recent hypotheses that increased bisretinoid levels in the RPE cause increased stress and dysfunction in Stargardt’s macular dystrophy and age-related maculopathy, our ability to simultaneously map local changes in bleachable rhodopsin along with changes in bisretinoid (A2E) fluorescence may improve evaluation of these conditions. Hence, it may help better assessing early interventions to prevent progression of highly localized pathologies. By computing simpler fractional change maps between early and late cSLO bleaching image sequences, we have observed decreased bleachable rhodopsin at hyperfluorescent lesion edges in a central Stargardt’s lesion as well as decreasing bleachable rhodopsin as one approaches the hypofluorescent centers of each reticular druse. Using the more integrated analysis presented here, could make such clinical observations more precise and lead to better understanding the relationships between increased bisretinoids, RPE dysfunction, and local visual cycle deficiencies that led to rod dysfunction and loss.

## 8 Conclusions

We developed noninvasive multispectral retinal fluorescence imaging by adding filter sets to standard fundus cameras enabling us to compute spatial macular pigment maps. The macular pigment maps are then combined with 1-minute-long cSLO autofluorescence imaging sequences starting at a moderately dark-adapted state to create additional maps of rod rhodopsin and RPE bisretinoid fluorescence using a biophysical bleaching and regeneration model and mathematical optimization techniques. To foster future collaborations and synergies, we outlined both the biomedical and the mathematical approach in detail. Retinal blood vessels, blocking the 488*nm* laser beam, are removed from the rhodopsin maps by image inpainting. We computed spatial rod rhodopsin distribution maps that match the characteristics of rod distribution observed in the literature. Further efforts are required to derive quantitative rod rhodopsin density maps from our proposed approach. Our limitation to qualitative density maps is due to the necessity of background removal in our image sets, whose methodology we have not been able to validate up to now. Our modular image analysis approach is well-suited to incorporate additional analysis steps, so that a validated background removal methodology would enable spacial mapping of rod rhodopsin density in a quantitative fashion.

We derived so far a non-invasive clinical methodology to map local rod rhodopsin distribution at ≈ 50*μm* resolution using widely available clinical imaging devices. Our approach appears well-suited to identify localized changes in the rod photoreceptors and relate them to local changes within the underlying RPE bisretinoid fluorescence levels. Although this method appears robust in younger eyes and in elderly pseudophakes, we need further optimization of our methods for those elderly patients, in which age-related lens changes combine with spatially varying diffuse reflectance of the retina creating non-uniform backgrounds in our image sets. Additional refinement and validation may enable us in future studies to correlate local rhodopsin reductions and autofluorescence levels within early microscopic lesions in order to better understand their natural progression and their responses to benign disease prevention strategies that are most likely to be effective in such early disease states.
